# Knowledge of Healthcare Coverage for Refugee Claimants: Results from a Survey of Health Service Providers in Montreal

**DOI:** 10.1371/journal.pone.0146798

**Published:** 2016-01-20

**Authors:** Mónica Ruiz-Casares, Janet Cleveland, Youssef Oulhote, Catherine Dunkley-Hickin, Cécile Rousseau

**Affiliations:** 1 Department of Psychiatry, McGill University, Montreal, Quebec, Canada; 2 SHERPA-Institut Universitaire, Centre intégré universitaire de santé et de services sociaux du Centre-Ouest-de-l'Île-de-Montréal, Montreal, Quebec, Canada; 3 Harvard School of Public Health, Cambridge, Massachusetts, United States of America; 4 Center for Clinical Epidemiology and Community Studies, Jewish General Hospital, Montreal, Quebec, Canada; University of South Australia, AUSTRALIA

## Abstract

Following changes to the Interim Federal Health (IFH) program in Canada in 2012, this study investigates health service providers’ knowledge of the healthcare coverage for refugee claimants living in Quebec. An online questionnaire was completed by 1,772 staff and physicians from five hospitals and two primary care centres in Montreal. Low levels of knowledge and significant associations between knowledge and occupational group, age, and contact with refugees were documented. Social workers, respondents aged 40–49 years, and those who reported previous contact with refugee claimants seeking healthcare were significantly more likely to have 2 or more correct responses. Rapid and multiple changes to the complex IFH policy have generated a high level of confusion among healthcare providers. Simplification of the system and a knowledge transfer strategy aimed at improving healthcare delivery for IFH patients are urgently needed, proposing easy avenues to access rapidly updated information and emphasizing ethical and clinical issues.

## Introduction

“It is difficult to feel up-to-date on these issues. It seems to me that every year we receive new contradictory information.”

Survey respondent

Systemic barriers to health care access have been documented for both asylum seekers and resettled refugees in Canada [1–3]. In spite of their well-documented high medical and psychosocial needs [4–6], refugees frequently have difficulty obtaining adequate healthcare due to limited or no health insurance, low socioeconomic status, language barriers, lack of familiarity with host country’s systems, cultural differences, and discrimination [1,6–11]. Refugee claimants and failed claimants may be reluctant to seek care because of fears of a negative impact on their migration status [2]. Studies further suggest that migrants who face refusals of care or demands for fees may subsequently hesitate to seek care [9]. In Canada, there is evidence that refugee claimants covered by the Interim Federal Health (IFH) program have often been refused care or charged fees due to confusion among healthcare providers about the extent of IFH coverage [7,12] and the complexity of the reimbursement scheme [9].

The IFH program was established by the federal government in 1957 to provide refugees and refugee claimants (and their dependents) with access to medical services upon arrival in Canada if they were not yet covered by a provincial or territorial health insurance plan. Originally a Health Canada program (Order in Council P.C. 157-11/848 of June 20, 1957), the IFH has been delivered by Citizenship and Immigration Canada since 1995. Before 2012, IFH coverage was equivalent to provincial health insurance combined with social assistance health benefits, including almost all medical services and medications, as well as certain supplemental services, for all resettled refugees as well as refugee claimants until they were either accepted or, if rejected, until the date of removal.

Resettled refugees are recognized as refugees following an overseas screening process and receive permanent resident status before arrival in Canada. They may be sponsored either by the government or privately, in the latter case by non-profit organizations such as churches or by groups of individuals. During the first year they receive income support from the government or from their private sponsor. Refugee claimants, on the other hand, enter the country by their own means and claim refugee status on site. Their claims are heard by an independent tribunal, the Immigration and Refugee Board, which will determine whether they meet the legal criteria for recognition as refugees, including being at risk of persecution on grounds such as ethnicity, religion, political opinions, gender or sexual orientation if returned to their country of origin.

On June 30, 2012, the federal government reduced the scope of the IFH, so that only Government Assisted Refugees retained full coverage of medications. For all others, medications ceased to be covered except for conditions threatening public health or safety, defined as contagious diseases and aggressive psychotic disorders. Medical services continued to be fully covered for most refugee claimants. However, claimants from countries deemed safe by the Minister of Immigration (Designated Countries of Origin) and refused claimants lost all medical coverage except for public health and safety conditions. Designation of these countries took place from 2012 to 2013, significantly shifting the map of coverage. Prior to June 2012, IFH had offered a single basket of services for all categories of refugee claimants and resettled refugees. The new program divided the refugee population into eleven categories with different degrees of entitlement to the four baskets of services that were offered.

In Quebec, the provincial government immediately stepped in to compensate for gaps in federal coverage of medical services and medications for all refugee claimants and refused claimants with a valid IFH certificate. As a result, claimants and refused claimants remained entitled to the same coverage as a person with provincial health insurance (RAMQ) with a few rare exceptions (e.g., organ transplants). Quebec also provided coverage of certain supplemental services such as rehabilitation and home care. Physicians were instructed to first bill Medavie Blue Cross (which administers the federal IFH program) and, in case of refusal, to submit the bill to the RAMQ. Hospitals and public clinics were to shoulder institutional costs. The Quebec Ministry of Health explicitly prohibited physicians and health institutions from charging fees to patients presenting with a valid (non-expired) IFH certificate.

On November 5, 2014, following a Federal Court decision declaring that the 2012 IFH cuts violated the Canadian Charter of Rights, the federal government adopted a new, temporary version of the IFH which reinstates some, but not all, of the pre-June 2012 IFH benefits [13]. For example, most Privately Sponsored Refugees, refugee claimants and refused claimants still have no coverage of prescription medication except for Public Health–Public Safety conditions, unless they are pregnant women or children under 19. Coverage of supplemental services is even more limited. The 2014 version of the IFH remains complex, with thirteen categories of insured refugees and five baskets of services.

This survey was conducted before these latter changes. In principle, claimants and refused claimants in Quebec have continuously been entitled to broad coverage of medical services and medications. However, confusion among health service providers and staff about the scope of coverage, onerous billing procedures, fears of nonpayment and incorrect assumptions that services are not covered, appear to have contributed to healthcare access problems. The objective of this study is to assess Quebec healthcare providers’ knowledge of the provincial and federal health care coverage for refugee claimants, and to analyze the associations between knowledge and personal and institutional factors.

## Method

### Study population

All administrators, clinicians, and other staff in five hospitals and two primary care centres providing services to a majority of the Montreal population were invited to complete an online questionnaire. Montreal was selected as site of the survey given that it is the largest settlement location in Quebec and the second largest in the country [14]. Of the 2,065 people who initiated the survey, only those who completed it were included in the analysis (n = 1,772).

### Survey Design and Administration

Refugee claimants (aka asylum seekers) were defined as individuals who apply for refugee status in Canada until either they are accepted as refugees or they are deported following the definitive rejection of their refugee claim. They are legally in Canada and entitled to IFH coverage. Government Assisted and Privately Sponsored Refugees eligible for provincial health insurance upon arrival were not considered in the study.

A questionnaire was designed by a multidisciplinary team of health and social service providers, translated (from English to French), and pretested. The survey instrument contained 18 multiple-choice questions and one open-ended question [to elicit general comments on the subject.] Close ended questions recorded respondent’s (a) demographics, professional occupation, affiliation and exposure to refugee claimant clients; (b) knowledge about applicable federal and provincial policies on access to care for refugee claimants; and (c) opinions (i.e., extent of dis-/agreement with listed reasons for maintaining/expanding or restricting refugee claimants’ access to publicly funded health care) and (d) practices regarding healthcare [services] for refugee claimants (e.g., “if a refugee claimant seeking care at my health institution does not appear to have health insurance coverage, I usually …” [list of possible behaviors provided for respondent to select all that apply]). The first two survey sections were the same for all respondents and are the focus of this manuscript. The three knowledge questions were presented by increasing order of difficulty. The first question was designed to test knowledge of the basic premise of the program, i.e., that in Quebec, refugee claimants with a valid IFH have the same coverage as RAMQ patients in most cases. Failure to grasp this principle indicates a fundamental lack of understanding of the program. The other two questions were probing for a more comprehensive knowledge of the coverage. The second question asked whether refused claimants lose all health care coverage. The final question assessed whether respondents knew that, in order to receive health services, refugee claimants whose IFH certificate has expired must pay and cannot claim reimbursement even if they renew their IFH certificate. Balanced wording was used to avoid bias. Only one response choice was allowed per question; all questions had a “Don’t Know” option although respondents were encouraged to answer to the best of their knowledge.

The questionnaire was administered using LimeSurvey, an open source survey application, over a period of six consecutive weeks in May-June 2014. A link to the survey was posted on the institutions’ intranet and/or emailed to respondents (e.g., using physicians’ email list or Lotus Notes) and individual email reminders were sent twice during the survey period.

Participation was informed, voluntary, and anonymous. No question was mandatory except for the respondent’s gender, age, occupation, and institutional affiliation. A prize (one IPad) was drawn among respondents using a distinct registration system to preserve anonymity. Multicentric ethics approval was issued by the Research Ethics Board (REB) of the Centre de santé et de services sociaux (CSSS) de la Montagne, following approval by the REBs of: Jewish General Hospital, Centre hospitalier de l'Université de Montréal, McGill University Health Centre, Hôpital Maisonneuve-Rosemont, St. Mary's Hospital, and CSSS Bordeaux-Cartierville-St.-Laurent.

### Statistical Analysis

We used Chi-squared tests to assess univariate associations between levels of knowledge and personal and institutional factors. We considered age (≤ 29/30-39/40-49/50-59/≥60 years), gender (woman/man/other), language (French/English), institution (hospital/ primary care centre), immigration background or “generation status” (1^st^/2^nd^/3^rd^), previous contact with refugees (No/Yes), and contact with patients from different cultural backgrounds (very often or often (i.e., at least once a week) versus sometimes, rarely, never, N/A) as possible covariates in our multivariate models. We selected the covariates retained in the final models *a priori* using Directed Acyclic Graphs (DAG) [15].

The outcomes for knowledge were binary (correct/incorrect responses). We used log-binomial regressions to estimate the adjusted prevalence ratios (PRs) of correct responses in relation to individual and institutional characteristics. A dichotomized outcome with the number of correct responses in the three knowledge questions (< 2 vs. ≥ 2 correct responses) was constructed to that end. Because of the small number of missing data (n≤14 for any variable of interest), all the analyses were conducted on a complete case basis. We set the threshold for statistical significance at p<0.05 for main effects using 2-sided tests. All analyses were performed using R software [16]. Direct quotes from responses to the open-ended question are presented in italics to illustrate salient results.

## Results

### Characteristics of Respondents

The characteristics of the study population are presented in Table 1. Overall, 67% of medical doctors were specialists (vs. family doctors) (data not shown). The same proportion of respondents had contact with culturally diverse populations (76%) and were born in Canada (77%), although 21% of the latter had one or both parents born outside of the country. Contact with refugees varied across occupational groups (p <0.0001), with 61% of health care professionals, 36% of managers, 35% of administrative employees, and 33% of others indicating ever having contact with a refugee claimant seeking healthcare (Table 2).

**Table 1:** Demographic characteristics of the study participants (n = 1,772).

Characteristic	n	%
**Gender**		
Man	406	22.9
Woman	1361	76.8
Other	5	0.3
**Age**		
29 or younger	213	12
30–39	454	25.6
40–49	463	26.1
50–59	481	27.1
60 or over	161	9.1
**Generation**		
1^st^	409	23.3
2^nd^	364	20.7
Both parents born in Canada	985	56
Missing	14	-
**Occupation**		
Medical Doctor	332	18.9
Nurse	324	18.4
Social worker	116	6.6
Other professional	157	8.9
Administrative employee	463	26.3
Manager	206	11.7
Other	163	9.2
Missing	9	-
**Type of institution**		
Hospital	1362	76.9
Primary care centre	410	23.1
**Contact with refugee claimant**			
No	913	51.5
Yes	859	48.5
**Contact with culturally diverse population**			
≥ 1 per week	1352	76.4
< 1 per week ^a^	417	23.6
Missing	3	-

Note

_a_ Includes respondents who have no direct contact with patients.

**Table 2:** Contact with refugee claimants by profession of study participants.

		Contact with refugee claimants	
	Total N	N	%
**Occupation**			
Medical Doctor	332	226	67.7
Nurse	324	183	56.5
Social worker	116	92	79.3
Other professional	157	68	43.3
Administrative employee	463	161	34.8
Manager	206	74	35.9
Other	163	53	32.5

### Current Levels of Knowledge by Types of Respondents

Overall, levels of knowledge about refugee claimants’ healthcare coverage were very low with only 2% of respondents answering successfully all knowledge questions and 39% not giving the correct answer to any of them (Table 3). The proportion of correct responses was inversely related to the complexity of the question. Thus, 42% of respondents provided a correct response regarding access to health care coverage for refugee claimants with a valid IFH certificate (Question 1), whereas 24% of respondents provided a correct response regarding health care coverage for refused refugee claimants (Question 2), and only 15% of respondents provided a correct response regarding access to health care coverage for a refugee claimant with an expired IFH certificate (Question 3). Indeed, several respondents indicated in the final open-ended question knowing very little (if anything!) about “*the limits or restrictions to the IFHP*”, which was described as a complex program that was not easy to understand. Moreover, respondents indicated how “*since the new program is in effect it is increasingly difficult to know what kind of coverage are refugees entitled to*,” and acknowledged that this information is poorly transmitted in the healthcare system. For example, one respondent complained his institution “*has no information nor administrative support to guide physicians in this process*.” Respondents eloquently described how “*ignorance among healthcare providers in Quebec about what is covered by the IFH program causes much distress to beneficiaries regularly*.*”* Furthermore, “*there are too many CLSCs* [Local Community Health Centres, Centres locaux de services communautaires for the term in French] *and medical clinics that refuse to treat an asylum seeker even when s/he appears with a valid IFH* [certificate].”

**Table 3:** Frequency and percentages of correct responses by question and in aggregate.

	n	%
**Individual questions**		
Q1 ^a^	744	42.1
Q2 ^b^	429	24.3
Q3 ^a^	264	14.9
**Aggregated questions** ^c^		
3	33	1.9
2	287	16.2
1	764	43.1
0	688	38.8

Notes

_a_ n = 1768

_b_ n = 1765

_c_ n = 1772

Levels of knowledge varied by respondents’ characteristics (Table 4). There were significant differences in the percentage of correct responses by occupational group, with medical doctors and nurses having the highest percentage of correct responses in the first question (*P*_Q1_ = 0.01), yet social workers exhibiting better knowledge in the other two questions (*P*_*Q2*_ = <0.001; *P*_Q3_ = 0.003). Respondents who reported previous contact with a refugee claimant had significantly higher percentage of correct responses on the knowledge questions compared to respondents that reported no contact. Respondents who were first-generation immigrants, worked in primary care centres, or had more frequent contact with patients from different cultural backgrounds, had significantly higher percentage of correct responses for some questions but not for all.

**Table 4:** Univariate associations between percentage of correct responses and respondents’ characteristics by knowledge question.

Covariate	Q 1		Q 2		Q 3			
	% correct	p-value	% correct	p-value	% correct	p-value	% ≥2 correct	p-value
**Gender**		0.56		0.24		0.82		0.54
Man	43		26.5		15.8		18.7	
Woman	41.9		23.8		14.7		17.9	
Other	20		0		20		0	
**Age**		0.08		0.27		0.11		0.02
29 or younger	33		19.4		17		13.2	
30–39	42.8		23.2		12.4		17.4	
40–49	44.2		27.2		18		22.3	
50–59	42.6		24.6		14.6		18.3	
60 or older	42.1		24.8		11.9		13.7	
**Generation**		0.08		0.03		0.18		0.47
1^st^	46.8		28		17.7		20.1	
2^nd^	40.5		26.7		15.1		18.1	
Both parents born in Canada	40.6		22		13.8		17.3	
**Occupation**		0.01		<0.001		0.003		<0.001
Medical doctor	46.7		20.9		14.2		15.9	
Nurse	48.3		27.9		10.8		20.7	
Social worker	40		44.4		27.6		34.5	
Other professional	31.9		24.8		15.9		17.8	
Administrative employees	40.6		22		14.7		15.1	
Managers	41.3		21.1		16		15.3	
Others	38		20.9		14.1		18.4	
**Type of institution**		0.97		0.11		0.001		0.001
Hospital	42.1		23.4		13.5		16.4	
Primary care centre	42.2		27.3		19.9		23.7	
**Contact with refugee claimant**		<0.001		<0.001		0.02		<0.001
No	34.7		18.4		13.1		12.4	
Yes	49.9		30.6		16.9		24.1	
**Contact with culturally diverse population**		0.02		0.002		0.53		0.002
≥ 1 per week	43.6		26.1		15.2		19.7	
< 1 per week ^a^	37.2		18.6		13.9		13	
**Language**		0.77		<0.001		0.42		0.14
French	41.8		21.7		14.5		17.1	
English	42.6		29.6		15.9		20	

Note

_a_ Includes respondents who have no direct contact with patients.

Table 5 describes the results of the multivariate associations of the main covariates with the number of correct responses on the three knowledge questions. There was a significant association between knowledge and occupational group, age, and previous contact with refugees. For occupational group, social workers were significantly more likely to have 2 or more correct responses than doctors (PR = 1.91; 95% CI: 1.31, 2.76; *P* = 0.001). Likewise, respondents in the age-group 40–49 years were significantly more likely to have 2 or more correct responses than respondents in the younger age-group (PR = 1.59; 95% CI: 1.08, 2.34; *P* = 0.02). Finally, respondents who reported previous contact with refugees seeking healthcare were more likely to have 2 or more correct responses than respondents with no previous contact (PR = 1.84; 95% CI: 1.45, 2.30; *P* = <0.001).

**Table 5:** Multivariate associations between aggregated responses on knowledge questions and potential determinants.

Knowledge			95% CI	
	PR	p-value	Lower	Upper
**Occupation**				
Doctors	Ref.	-	-	-
Nurses	1.25	0.20	0.89	1.74
Social workers	1.91	0.001	1.31	2.76
Other professionals	1.29	0.25	0.84	1.97
Managers	1.06	0.79	0.70	1.61
Administrative employees	1.14	0.45	0.82	1.59
Others	1.39	0.11	0.92	2.10
**Age**				
29 or younger	Reference	-	-	-
30–39	1.21	0.33	0.82	1.80
40–49	1.59	0.02	1.08	2.34
50–59	1.31	0.18	0.88	1.94
60 or older	1.01	0.98	0.60	1.69
**Institution**				
Hospital	Reference	-	-	-
Primary care center	1.22	0.09	0.97	1.53
**Generation**				
1^st^	Reference	-	-	-
2^nd^	0.90	0.45	0.67	1.19
Both parents born in Canada	0.86	0.19	0.68	1.08
**Contact with refugee claimants**				
No	Reference	-	-	-
Yes	1.84	<0.001	1.45	2.30

## Discussion

Our study examined clinicians’ and administrative staff’s knowledge of healthcare coverage for refugee claimants following the federal government’s 2012 amendments to the IFH program through an online survey conducted in several Montreal hospitals and primary care centres. Overall, levels of knowledge about refugee claimants’ healthcare coverage were very low with only 2% of respondents answering successfully all knowledge questions and 39% not giving the correct answer to any of them. Social workers and respondents in the age-group 40–49 years were significantly more likely to know the coverage provided by the IFH program. Direct contact with refugees was associated with more knowledge.

The low levels of knowledge documented in our study suggest that the recent reforms to the IFH program may have generated or increased confusion among health care providers in navigating the revised IFH program. This confusion may lead to refusal of services to individuals with valid IFH coverage, requesting payment for services to which they are entitled or discouraging clinicians from providing services. Indirect negative consequences are also to be expected as refusal of care and demands of fees may deter refugee claimants from seeking health care in the future [9,17]. Poor knowledge uptake concerning the IFH program may be linked to an overly complex program, poorly designed implementation procedures, inadequate dissemination of information, and/or the target audience’s lack of interest (for example, busy physicians who experience difficulties being reimbursed may choose to refuse IFH patients rather than seeking additional information)[18]. On its face, the 2012 IFH was a complicated program, with its eleven categories of insured patients and four baskets of services. The Quebec government’s decision to compensate the gaps in federal coverage added another layer of complexity, in particular because of the two-step billing process. Respondents’ lack of understanding may also be due to insufficient information and inadequate approaches for disseminating information within the health systems, as well as for developing simple procedures to operationalize the IFH program (e.g., verification of coverage, billing, etc.) In Canada, the administrative complexity of the system and the deficiencies in dissemination of information about the 2012 IFH have been widely criticized by health service providers [19–22]. The logic of the 2012 scheme was particularly difficult for health service providers to grasp because it was designed to address migratory policy concerns rather than the healthcare needs of the refugee population [20–22].

Significant associations of knowledge with personal and professional variables suggest that the disciplinary perspective with refugee claimants may influence the level of knowledge of service providers. Of all healthcare service providers, it is not surprising that social workers are more familiar with the IFH program and its recent amendments as they are trained to be sensitive to social predicament and to work closely with refugee individuals and families to help them navigate the health system and connect with local resources. However, uncertainty about what refugee claimants are entitled to remains problematic for a large proportion of service providers across all occupational groups. The association between direct contact with refugee claimants and the level of knowledge could be explained by the effect of experiential learning which has been repeatedly described in the cultural competence literature [23]. It is also possible that this association reflects rather the fact that a proportion of clinicians who see refugees do so because they have a more positive attitude toward them or toward vulnerable population in general, and that this positive attitude is associated with better knowledge, or that those with better knowledge are more likely to see refugee patients (e.g., they might be more assured of reimbursement based on their knowledge that the provincial government will compensate gaps in federal coverage).

Results from this study need to be interpreted in light of some limitations. Variation in the means used for recruitment (i.e., physicians’ email list, intranet, Lotus Notes) may explain differential response rates by institution and professional groups. Response rates cannot be calculated due to lack of precise information about the number of individuals who received an invitation to participate in every institution. Although response rates by socio-demographic groups may parallel provincial figures for the health sector (e.g., 79% of employees are women), imbalanced participation of different sub-groups of respondents calls for caution in the interpretation of findings. Variation in computer literacy and strength of opinions on the issue under study may have contributed to differential response rates among some categories of respondents, thus limiting the generalizability of results. Refusal by some institutions to distribute the survey in both English and French may have forced some respondents to participate in a language they felt less comfortable in. Finally, inferences of causality are limited by the cross-sectional nature of our survey.

Despite these limitations, this study provides evidence of the need for targeted and updated knowledge transfer on health care access in times of administrative confusion and for further studies to inform the development of effective knowledge mobilization efforts on the IFH program and amendments. To be effective, it is essential to work at multiple levels, including simplification of the healthcare coverage program and its implementation as well as improved dissemination of information. It is advisable for those initiatives to provide information about the refugee claimants’ entitlement to health care in simple yet complete terms and to help the clinicians and the institutions navigate the billing system. In addition it may be appropriate to develop ethical and clinical guidelines which support clinician reflection and decision making, acknowledging that these may reflect their personal values, their professional code of ethics and the recommendation of their professional association among other [24,25]. It is important that any such initiatives target and reach different occupational groups (e.g., training clinicians as well as administrative and management staff), with a view at improving healthcare access and delivery for IFH patients in Quebec and across the country.
